# Why flowers close at noon? A case study of an alpine species *Gentianopsis paludosa* (Gentianaceae)

**DOI:** 10.1002/ece3.8490

**Published:** 2022-01-15

**Authors:** Qinzheng Hou, Xiang Zhao, Xia Pang, Meiling Duan, Nurbiye Ehmet, Wenjuan Shao, Kun Sun

**Affiliations:** ^1^ College of Life Sciences Northwest Normal University Lanzhou China

**Keywords:** floral closure, flower microenvironment, *Gentianopsis paludosa*, reproductive success

## Abstract

Repeatable floral closure with diurnal rhythms, that is, flower opening in the morning and closing in the evening, was widely reported. However, the rhythm of flower opening in the morning but closing in the midday received much less attention. *Gentianopsis paludosa*, Gentianaceae, has an obvious petal movement rhythm opening in the morning and closing at noon at northeast of the Qinghai‐Tibetan Plateau. In this study, we examined the effects of temperature (T), relative humidity (RH), and illumination intensity (II) on *G*. *paludosa's* flower closure. Furthermore, we monitored the environmental changes inside and outside of the flowers, aiming to test the effect of floral closure on the stability of microenvironment inside the flower. Finally, we artificially interrupted temporal petal closure and investigated its effects on reproductive fitness. The results showed that high/low temperature contributed more to the flower closure than low RH, while illumination intensity had no significant effect on it. The medium temperature, relative humidity and illumination intensity (environmental conditions at 10:00) did not delay flower closure when flowers at pre‐closing period or stimulate reopen when flowers full closed. Floral closure provided a stable temperature condition and a higher RH condition inside the flower. Meanwhile, compulsive opening and delayed closure of flowers decreased the seed‐set ratio while no effect was found when flowers were forced to close. We conclude that endogenous rhythm regulates floral closure. The rhythm of petal movement providing a stable microenvironment for *G*. *paludosa*, increasing the seed production and saving energy from flower opening maintenance, which might be an adaptive strategy to against unfavorable environmental conditions.

## Graphical abstract

In this work, we report a repeatable floral closure rhythm, that is, open in the morning but close in early afternoon, first in an alpine species belonging to Gentianaceae. By monitoring the petal movement under treatments of changed environmental factors, detecting the microenvironment changing inside and outside flowers, and measuring the seed production, we found that endogenous rhythm regulates floral closure, but which was also strongly influenced by high/low temperature. The floral closure at noon provides a stable microenvironment inside the flower from possible damage of both high and low temperature. Interestingly, high temperature could promote a rapid floral closure, which is a new finding because low temperature, rather than the high temperature, is assumed as the key limiting environmental factor. Besides, we found that floral closure at noon could assure the seed production. We conclude that floral closure at noon of *Gentianopsis paludosa* might be an effective adaptive strategy to against unfavourable environmental conditions. This is the first report of this petal movement rhythm of Gentianaceae as we know.

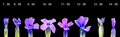

## 1 INTRODUCTION

The rapid movement of plants has been studied since Darwin (1880), and it is estimated that at least 1000 angiosperms show some form of repeatable movement in petals, carpels, or stamens (Simons, 1992). As one of the most extensively observed nonmorphological changes of flowers, the closure of petals, as well as the proximate underlying mechanisms, received strong attention (reviewed by van Doorn & Kamdee, 2014; van Doorn & van Meeteren, 2003). von Linné (1783) suggested that flower closure/opening follows fixed circadian rhythm (Linné’s clock), but, actually, various regulatory mechanisms or internal circadian rhythm were found to influence single flower closure. It is believed that the time of flower closing is likely an important evolutionary feature resulting from plant's fitness accrual (Ashman & Schoen, 1994).

Certain species close petals only once (e.g., Sigmond, 1929, 1930), while some others manifest repetitive petal closure and opening (e.g., Abdusalam & Tan, 2014). Although in some plant species, petal closure is apparently independent of specific external regulation as it occurs at any time of the day, in most species petal movement is repeatable showing a relationship with the time of day, such as day‐blooming flowers (close flowers at dusk or night, i.e., nyctinasty) and night‐blooming flowers (close flowers at dawn), which are widely reported and explored (reviewed by van Doorn & Kamdee, 2014; van Doorn & van Meeteren, 2003). Most of the previous works pointed out that opening and closure of flowers was regulated by several suspected regulatory mechanisms, such as internal circadian rhythm (Trivellini et al., 2016; Yon et al., 2016), light (Bai & Kawabata, 2015; Trivellini et al., 2016), temperature (Calinger et al., 2013; He et al., 2006), moisture (Magalhaes & Angelocci, 1976; Peter et al., 2004; Von Hase et al., 2006), and endogenous hormone (Ke et al., 2018). For example, the flowers of *Gentiana straminea* will close when decreasing temperatures approached 20 ℃ and subsequently began to reopen the following day when air temperatures warmed to approximately 13–15℃ (He et al., 2006). Additionally, some works pointed out that a rather complicated interaction exists between the influence of different exogenous (e.g., pollinators, light intensity, humidity) or endogenous factors (e.g., genetically programmed, sexual specialization, hormonal regulation) (Berjano et al., 2014; Ferval et al., 2013; Prokop et al., 2021; Prokop & Neupauerova, 2014).

Among the flowers that show repeated opening and closure, mostly opened during the day and closed during the night, whereas some species close during a specific time of the day. For example, *Tragopogon pratensis* closes early in the morning, *Sonchus arvensis* and *S*. *oleraceus* close at midday, and some other species close early in the afternoon (Brauner & Rau, 1966; Han et al., 2021). This kind of flower movement is relatively rare in the field and seldom reported (most in Asteraceae and a few cases in Primulaceae. Brauner & Rau, 1966). As the weather factors always fluctuate throughout the day, it provides a clue that the intermittent flower closure may be influenced by maximum and/or minimum environmental condition(s). Flower closure may provide a relatively stable internal environment to avoid the effects of weather fluctuations. Moreover, flower opening and closure can partially reduce flowering costs (van Doorn & van Meeteren, 2003), which can occur repetitively or only once. It has been proposed that repetitive closure may be a compromise between maximizing reproductive success and minimizing the costs of flower maintenance (Ashman & Schoen, 1996; Schoen & Ashman, 1995). For species with a floral movement rhythm of opening in the morning but closing at noon, reduced opening time possibly decreases reproductive fitness because of decreased opportunity for outcrossing induced by shortened flower representation time to pollinators. However, there are few studies focused on the puzzling phenomenon and the evolutionary processes underlying this phenomenon are largely unknown.

*Gentianopsis paludosa* belongs to Gentianaceae and usually grows in alpine regions. At northeast Qinghai‐Tibetan Plateau (QTP), this species flowers from early July to late August, which is the growing season for most species. Based on previous observations at northeast QTP, we found no effective pollinators of *G*. *paludosa*, which is consistent with previous reports that the visitors of *G*. *paludosa* were extremely rare in high‐altitude populations (Duan et al., 2010). Importantly, we found that the flowers of *G*. *paludosa* opened in the morning, but closed at noon, and a full‐closure completed at early afternoon. Although repeatable floral closure is commonly seen in Gentianaceae, mostly are nyctinasty species, such as *Gentiana straminea* (He et al., 2006), *Gentiana algida* (Bynum & Smith, 2001), *Gentiana leucomelaena* (Mu et al., 2010), and *Eustoma grandiflorum* (Bai & Kawabata, 2015). An intermittent flower closure of Gentianaceae, which opens in the morning and closes at noon, is never reported. As the minimum relative humidity, maximum temperature, and illumination intensity always occurred at about 14:00, the environmental condition when the flowers of *G*. *paludosa* (12:00) begin to close was not the limiting factors during the day time. To test the hypothesis that repeatable flower closure is a strategy to ensure reproductive success by providing a relatively stable internal environment and saving energy from flower opening maintenance, and explore the ecological function of this phenomenon, we used *G*. *paludosa* as the research material and addressed the following questions: (1) whether changed ambient environment conditions would stimulate (or delay) flower closure? (2) whether closed flowers could re‐open in changed ambient environment conditions? and (3) what is the ecological function of the intermittent flower closure?

## 2 MATERIALS AND METHODS

### 2.1 Study species and area

**FIGURE 1:**
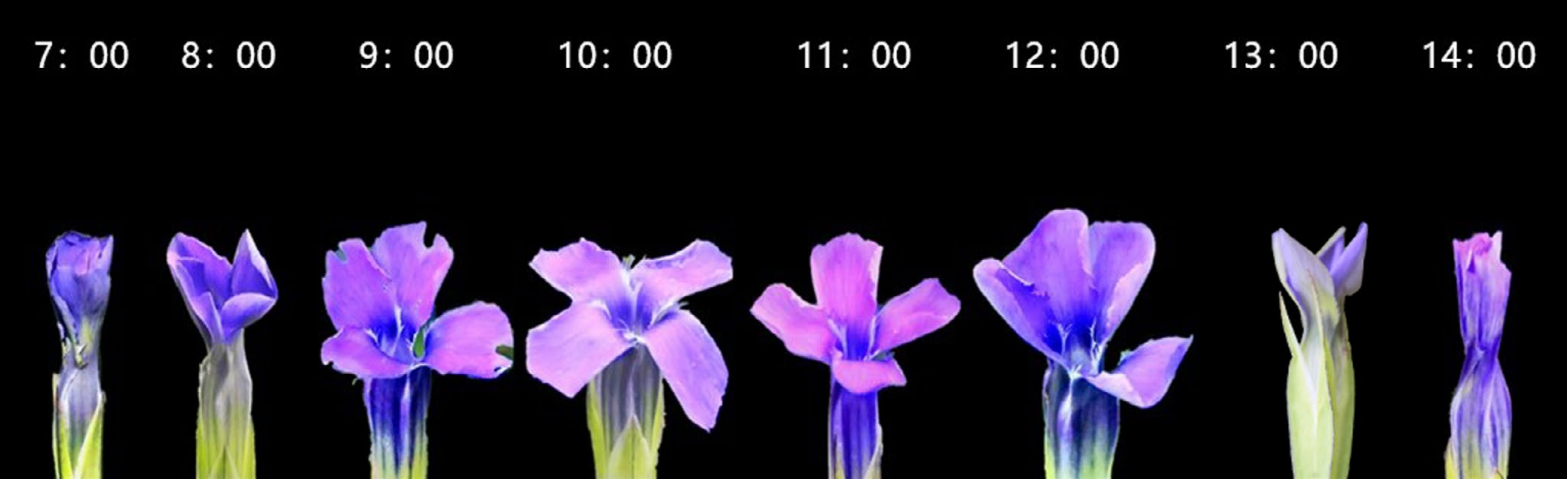
The process of floral opening and closure in *Gentianopsis paludosa*

*Gentianopsis paludosa* is a biennial herb of Gentianaceae, growing on the beaches, hillsides, grasslands, and forests (alt 1180–4900 m.a.s.l.) of alpine environments (Ho & Pringle, 1995). The plant blooms from early July until early August; ranges from 3.5 cm to 40 cm in height and carries several single terminal flowers on different stalks (Duan et al., 2010). At our study site, *G*. *paludosa* is fully self‐compatible, and the flower is about 4–5 cm in length and has a long corolla tube which is cylindrical and funnel‐shaped (Figure 1). No reliable pollinators were found in this study site, suggesting predominantly autonomous selfing in *G*. *paludosa*.

Our work was carried out between early July and late August from 2018 to 2020 in a natural population at the Alpine Meadow Ecosystem Scientific Research Station of Lanzhou University at Gannan, Gansu Province, China (longitude 102°53′E, latitude 34°55′N, altitude 2900 m). The climate is cold humid‐alpine with mean annual rainfall of 620 mm, and the mean annual temperature is 1.2°C with −10.7°C in January and 11.7°C in July. The vegetation consists mainly of alpine meadow and steppe (Wu, 1995) and is dominated by Cyperaceae, Gramineae, Compositae, and Ranunculaceae.

### 2.2 Floral movements and microenvironmental condition monitoring

**FIGURE 2:**
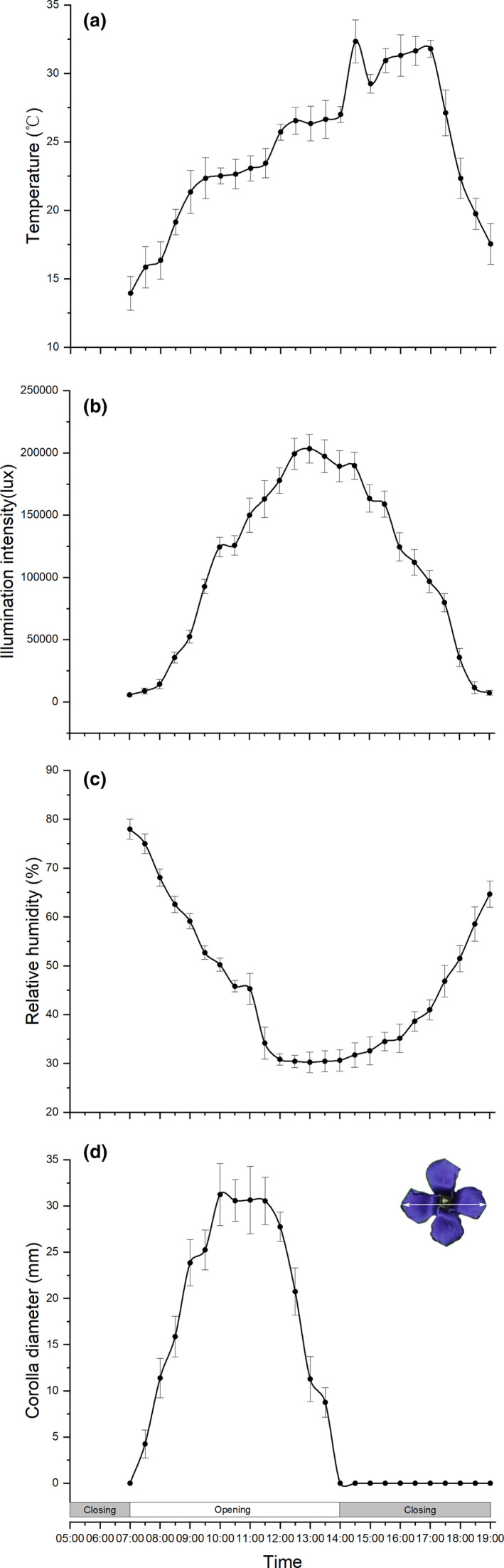
Diurnal environmental factors and corolla diameter variation in *Gentianopsis paludosa*. (a) Temperature. (b) Illumination intensity; (c) Relative humidity. (d) Corolla diameter. Bars indicate the standard deviations (*N* = 40)

The floral movements of *G*. *paludosa* were monitored in the full bloom stage for 2 consecutive years in the field (from 22 July to 10 August in 2018 and from 16 July to 5 August in 2019). During each time period, sunny days were selected to conduct the floral movement monitoring. Every half‐hour, from the prebeginning of bloom (7:00) to postdusk (19:00), the flower outer diameter (the distance between opposite lobes, Figure 2d) of *G*. *paludosa* (*N* = 20 each year) were measured using vernier caliper to monitor the process of flower opening and closure.

At the same time of corolla width measuring, the microenvironmental climate conditions were also measured. An air temperature and relative humidity measuring instrument (AR847; XI’MA) was used to test the air temperature (T) and relative humidity (RH), and an illuminance meter (TES‐136; MINGBO) was used to test the illumination intensity (II) adjacent to individual corollas. The correlations among three environmental factors during the day were also analyzed to examine the relationship between them.

### 2.3 Floral movements caused by changing environmental conditions

**FIGURE 3:**
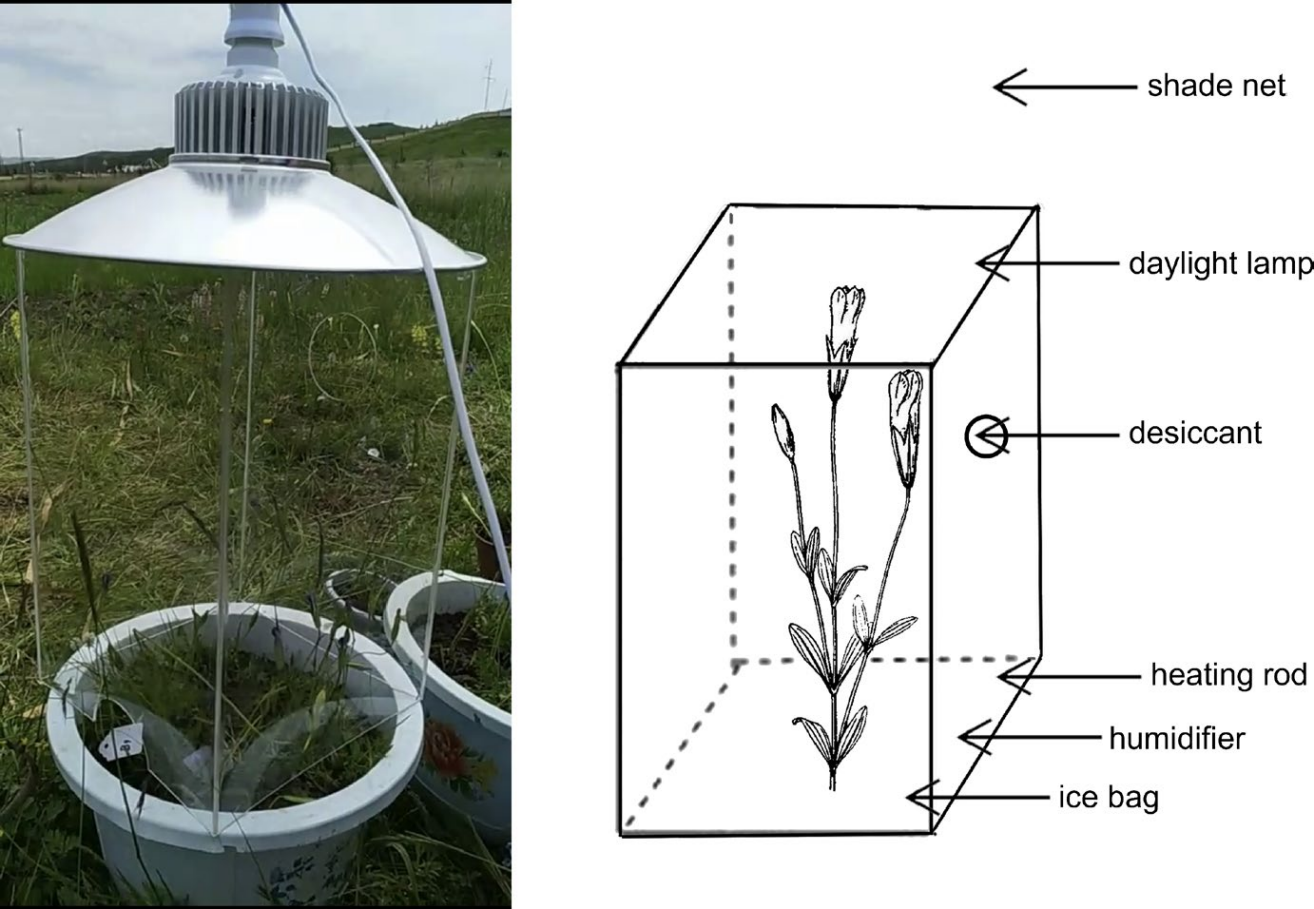
Diagram of control device for ambient environmental factor regulation and control

To test whether changed environmental conditions could influence floral movement, and, which environmental factors could influence floral movement, we conducted the experiments in the field in 2019 and 2020. A total of more than 50 individual plants, with flower buds, were excavated from the study population along with surrounding substrate to minimize disturbance. They were transported to the experimental garden near the station and individually planted in plastic flower pots (vol. 3.5 L). Each pot was watered and placed in an open, unscreened place. Experiments were conducted when the flowers opened on subsequent days. Some transparent acrylic covers (30 cm width, 30 cm length, and 60 cm height) with an empty bottom and some small holes on the side wall were made for ambient environmental factor regulate and control (Figure 3). The temperature was controlled by electrical heating rod (temperature increment) and ice bags (temperature reduction); the illumination intensity was controlled by thickness adjustable shade net (illumination intensity reduction) and daylight lamps (LICOR INC, Lincoln, Nebraska, USA) (illumination intensity increment); the humidity was controlled by adjustable humidifier (humidity increment) and desiccants (humidity reduction) (Figure 3).

Based on the results of floral movements in natural condition, we selected 90 flowers of full‐blooming period (10:00) and 90 flowers of closed period (14:00) to test whether changed ambient conditions would stimulate closing or re‐opening, respectively, conducted the following 9 treatments (10 flowers for each treatment) from then on, and recorded the corolla width per half‐hour: (1) closing T: keep the ambient temperature to 25 ± 0.5℃ (the temperature when flowers begin close at 12:00); (2) opening T: keep the ambient temperature to 14 ± 0.5℃ (the temperature before opening at 7:00); (3) blooming T: keep the ambient temperature to 22.5 ± 0.5℃ (the temperature of full‐blooming at 10:00); (4) closing II: keep the illumination intensity to 177,000 ± 1000 lux (the illumination intensity when flowers begin close at 12:00); (5) opening II: keep the illumination intensity to 5600 ± 800 lux (the illumination intensity before opening at 7:00); (6) blooming II: keep the illumination intensity to 130,000 ± 1000 lux (the illumination intensity of full‐blooming at 10:00); (7) closing RH: keep the RH to 30 ± 2% (the RH when flowers begin close at 12:00); (8) opening RH: keep the RH to 78 ± 2% (the RH before opening at 7:00); (9) blooming RH: keep the RH to 50 ± 2% (the RH of full‐blooming at 10:00).

To explore whether changed environmental conditions would delay the floral closure, 30 flowers of preclosing period (12:00) were selected and the following treatments were conducted (10 flowers for each treatment and the methods were the same as above): (1) blooming T; (2) blooming II; (3) blooming RH.

Furthermore, combination treatments were conducted in the field. Based on the methods mentioned above, we conducted all the 2‐factor‐combined treatments on full‐blooming period and closed period flowers, respectively (totally 12 combined treatments, *N* = 10, respectively). Considering that in the single‐factor experiment, the other two environmental factors were controlled at the conditions of full‐blooming period (10:00), so blooming T, RH, and II were not conducted in the 2‐factor‐combined treatment. Because one changed environmental factor would influence the other more or less (such as a temperature increase would happen accompanied by increased illumination intensity), although single factor and 2 combined factor treatments were easily controlled, we failed to conduct the 3 factor‐combined treatments.

For all the experiments, the continued ambient factors conducted on full‐blooming and preclosing period flowers lasted until flowers closed, and those conducted on closed period flowers lasted for 2 h.

### 2.4 Effect of floral closure on microenvironmental stability inside the flower

To explore whether floral closure provide a stable microenvironment condition inside the flower, the temperature, and relative humidity inside and outside the flower were tested. On sunny days, 15 flowers were randomly tagged each day, the temperature and relative humidity inside the flower were monitored by inserting a beaded temperature and humidity sensor (AR847; XI’MA) into the half depth of corolla, the temperature and humidity outside the flower were tested by placing the sensor to the outside of the corolla (at the same height and 2–3 cm away). This experiment was executed per hour after 6:00 (preopening) till 19:00 (postclosure) on sunny days from 20 July to 1 August, 2020. The temperature and humidity differences between inside and outside of flower at each time were compared using paired‐samples *T* test (SPSS 13.0). Coefficient of variation (CV) was used to evaluate the stability of temperature and humidity between inside and outside flower, while a small CV means a better temperature and relative humidity stability. Coefficient of Variation (CV) was calculated as CV = standard deviation/mean, which is a data dispersion parameter, and the mean value used in the formula is from all means of each time point.

### 2.5 Effect of floral closure on seed production

**FIGURE 4:**
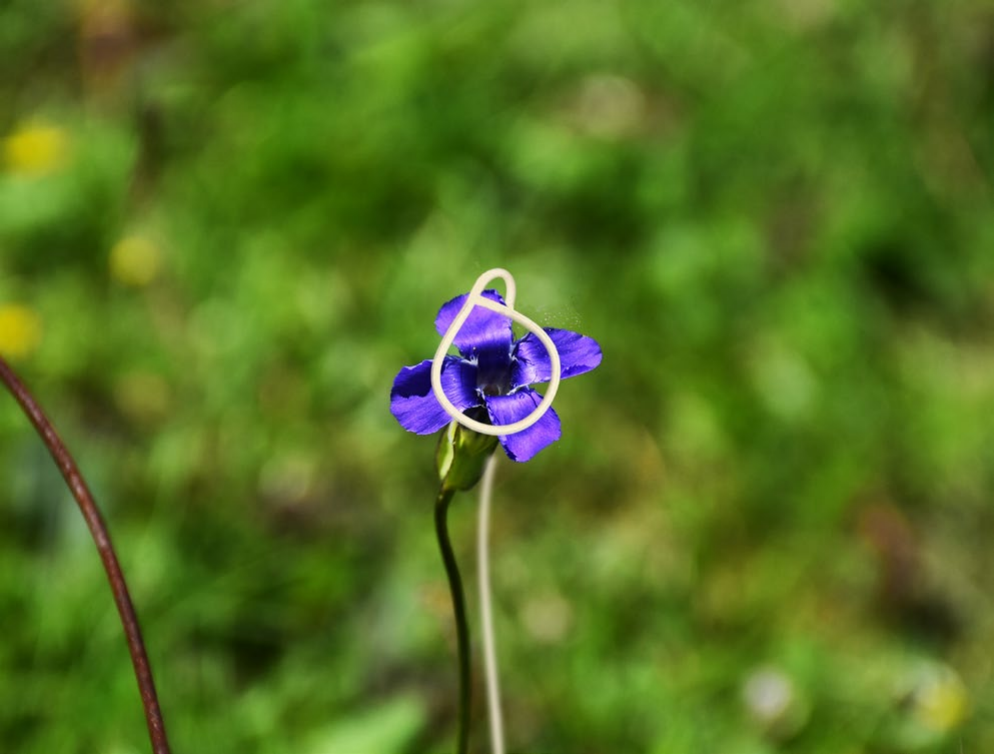
An example of artificially interrupted petal closure in the field

The effect of floral closure on seed production was tested in the field in 2019 and 2020. We tagged 120 individual flowers each year, 4 treatments were conducted (each for 30 flowers) when flowers opened. (1) a compulsive openness following Prokop et al. (2019) was conducted using a wire to prevent flower closure (Figure 4); (2) a forced closure following He et al. (2006) was conducted using transparent adhesive tape to keep flowers close; (3) a delayed closure was conducted using a wire to prevent closure when flowers open in the morning (7:00), but remove the wire at dusk (19:00); and (4) the flowers were kept naturally for a control group. We collected all the seeds when mature, and counted the seed‐set ratio (the seed set ratio was calculated as the ratio of the number of plump seeds to the total number of seeds per fruit). The data were analyzed by ANOVA to evaluate the main effects of different treatments on seed‐set ratio. All statistical analyses were carried out with SPSS for Windows, version 13.0 (SPSS Inc.).

## 3 RESULTS

### 3.1 Floral movements rhythm and microenvironmental condition

Flowers of *G*. *paludosa* began to open at about 7:00 in the morning, when the temperature was 13.94 ± 1.23℃, relative humidity was 77.98 ± 2.05% and the illumination intensity was 5629.5 ± 848.73 lux. The opening process lasted about 3 h till 10:00 reached to a full‐blooming, when the temperature, RH, and illumination intensity was 22.52 ± 0.57℃, 50.23 ± 1.32%, and 124,430.5 ± 7776.46 lux, respectively. The full‐blooming period lasted about 2 h, and after that, the flowers began to close (12:00), when the temperature, RH, and illumination intensity was 25.72 ± 0.59℃, 30.85 ± 1.19%, and 177,850.5 ± 10,168.49 lux, respectively. The closing process lasted about 2 h till 14:00, and the corolla became fully closed, when the temperature, RH, and illumination intensity was 27.01 ± 0.57℃, 30.65 ± 2.18%, and 189,299.5 ± 12,620.49 lux, respectively (Figure 2). There was a significant correlation between environmental factors within a day. Temperature was positively correlated with light intensity but negatively correlated with relative humidity (Table 1).

**TABLE 1:** Pearson's correlation coefficients (*r*) for the environmental factors during the day

	Temperature	Relative humidity	Illumination intensity
Temperature		−0.865**	0.723**
Relative humidity			−0.932**
Illumination intensity			

**p < .01.

*Gentianopsis paludosa* showed an obvious repeated flower opening and closure, representing a 7 h open and 17 h close rhythm, although, actually, the full‐blooming period was short (2 h). However, the repeatable opening and closure rhythm did not relate to any of the single environmental changing, since the peaking time of temperature, RH, and illumination intensity was at 14:30, 13:00, and 13:00, respectively (Figure 2).

### 3.2 Changed environment conditions influenced floral closure

**FIGURE 5:**
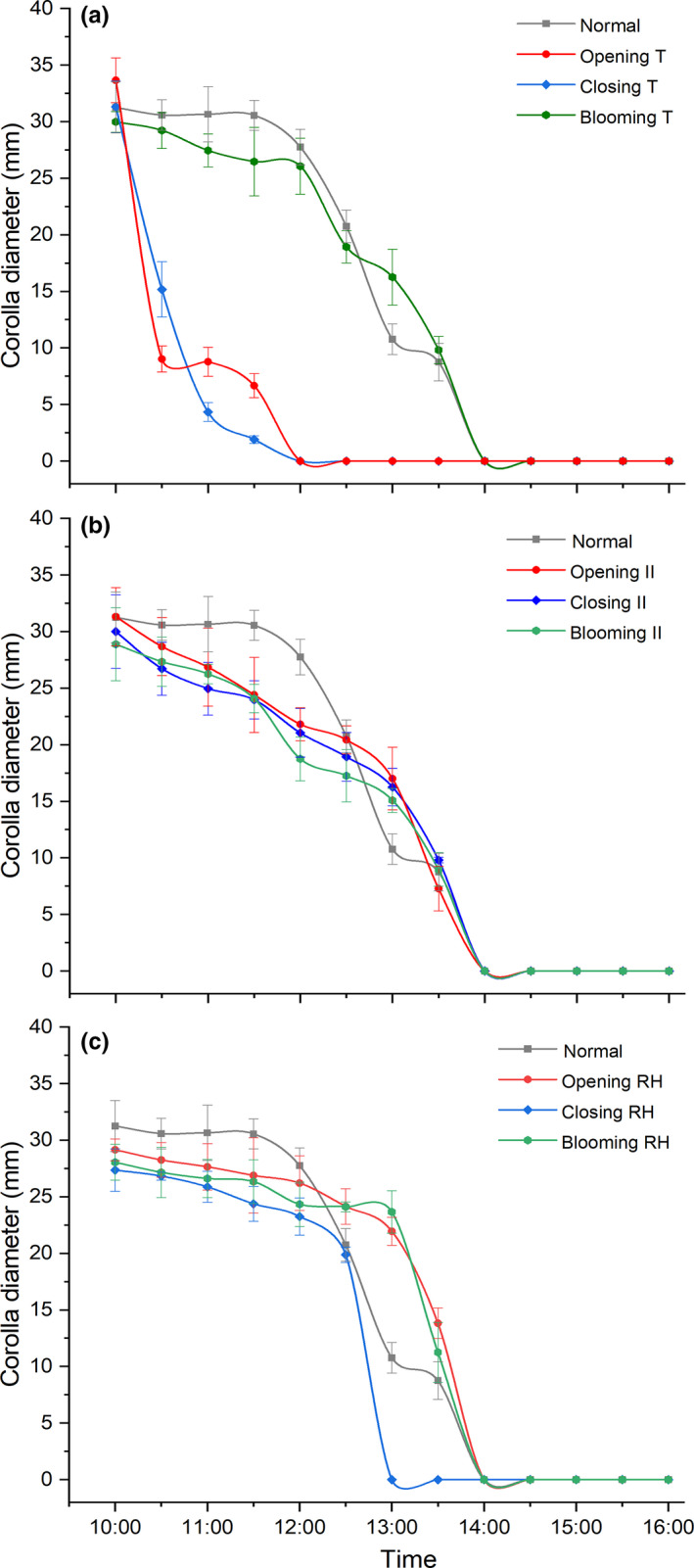
Effects of different levels of environmental factors on corolla diameter after full‐blooming period (10:00). (a) Temperature. (b) Illumination intensity; (c) Relative humidity. Bars indicate the standard deviations (*N* = 10). II, illumination intensity; RH, relative humidity; T, temperature. The same as below

When we conducted a low temperature (opening T) or high temperature (closing T) at the blooming period (10:00) of *G*. *paludosa*, the outer diameters of the flower would decrease in a short time (Figure 5a), indicating a rapid floral closure caused by both low and high temperatures, and flower closes 2 h earlier (both full closed at 12:00) compared with natural condition. Besides, the treatment of continued medium temperature (blooming T) did not advance or delay the flower closure of *G*. *paludosa* (Figure 5a), indicating that continued medium temperature had no influence to the floral closure of *G*. *paludosa*.

Contrast to a rapid floral closure and flowers close earlier caused by high/low temperature, the outer diameter of *G.* *paludosa* decreased gradually and fully closed at 14:00 under continued illumination intensity treatment (including high, low, and medium illumination intensity), which was similar to natural condition (Figure 5b). Continued high RH and medium RH could not delay or advance the time of floral closure (the full closure time was 14:00), but could slow the closing process, which was similar to the effect of changed illumination intensity (Figure 5c). However, continued low RH (closing RH) could advance the floral closure to 13:00, which was 1 h earlier than natural condition, and continued low RH could accelerate closing, inducing a rapid floral closure (Figure 5c).

**FIGURE 6:**
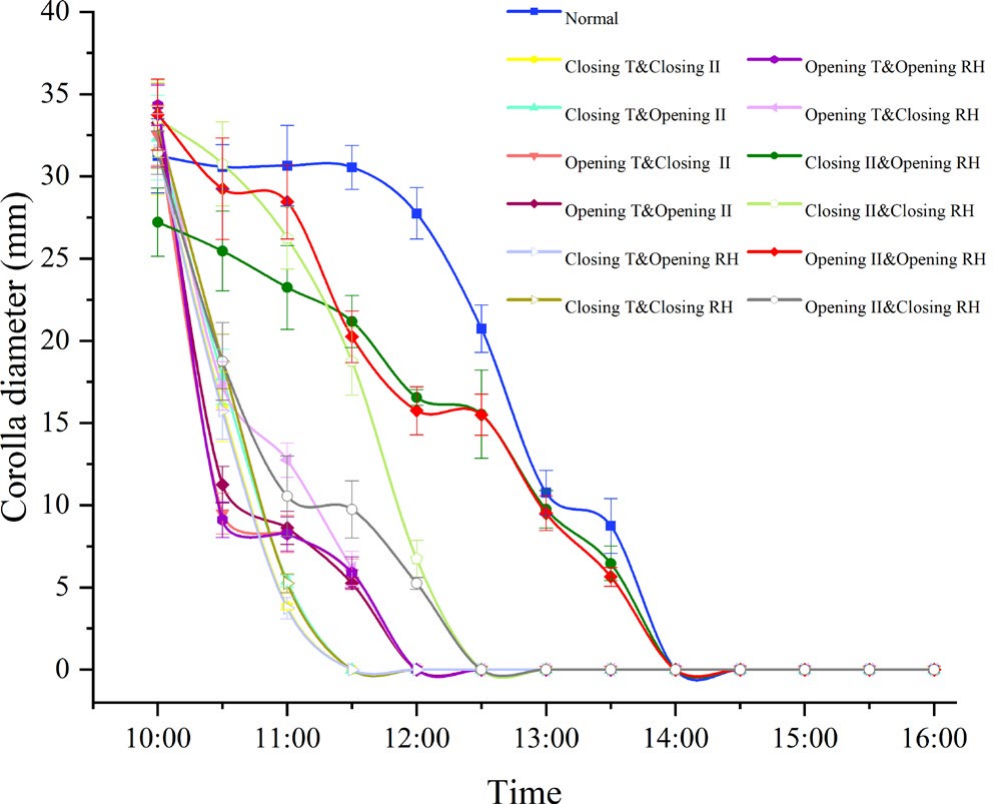
Effects of combined treatment of environmental factors at different levels after full‐blooming period (10:00) on corolla diameter. Bars indicate the standard deviations (*N* = 10)

When 2‐factor‐combined treatments conducted on full‐blooming flowers, all the temperature‐combined treatments could induce a rapid floral closure, especially the high temperature (closing T), which would stimulate flower close 2.5 h earlier compared with natural condition (flowers closed at 11:30), while the low temperature (opening T)‐combined treatments could stimulate flower closure 2 h earlier (flowers closed at 12:00, Figure 6). Low RH (closing RH)‐combined treatments, as well, could induce flower close 1.5 h (combined with illumination intensity) or 2–2.5 h (combined with temperature) earlier. However, high RH (opening RH) combined with high and low illumination intensities treatments did not accelerate flower closure, although the closing process became gradually (Figure 6).

**FIGURE 7:**
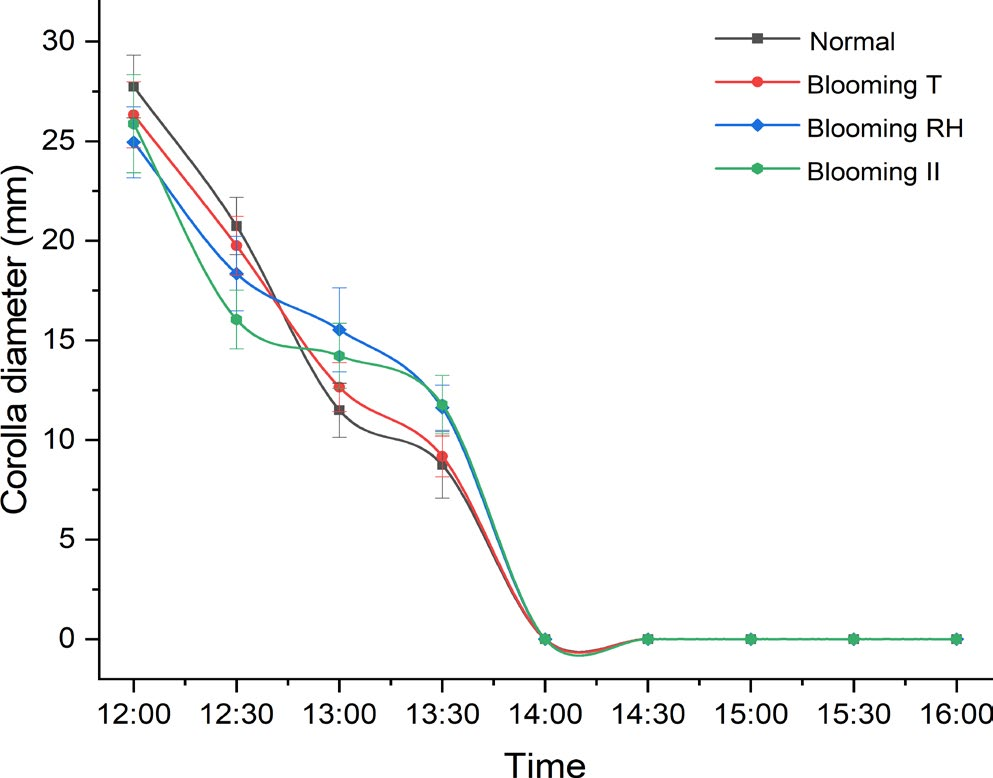
Effects of different environmental factors on corolla diameter after preclosing period (12:00). Bars indicate the standard deviations (*N* = 10)

When changing the temperature, RH, and illumination intensity to the blooming state, preclosing period (12:00) of the flowers showed a rapid floral closure, and a full‐closure occurred at 14:00, which had no difference with the natural condition (Figure 7). This result indicated that the medium environment conditions could not influence the closure process when the flowers at a preclosing period.

After a full closure, the flowers of *G*. *paludosa* did not re‐open again after any of the treatments, including all the single‐factor treatments and 2‐factor treatments.

### 3.3 Floral closure benefited microenvironment stability inside the flower

**FIGURE 8:**
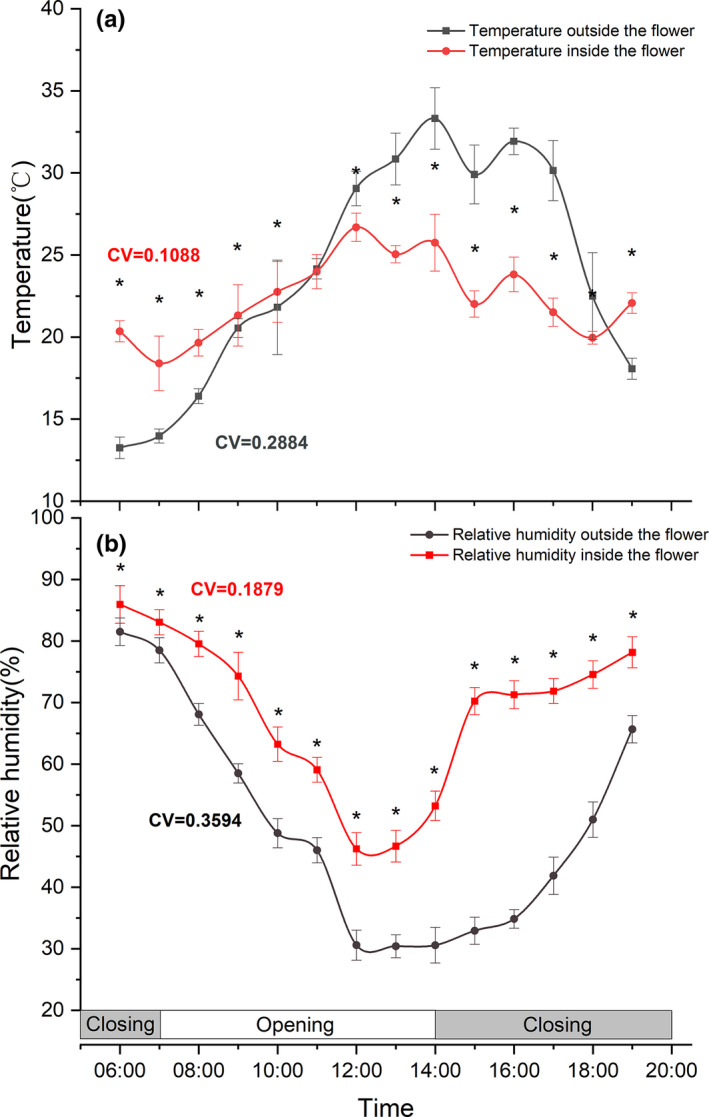
Temperature and humidity variation of outside and inside flowers of *G*. *paludosa*. Asterisks indicate a significant difference at 0.05 level. (a) Temperature. (b) Relative humidity. Bars indicate the standard deviations (*N* = 45)

The temperature inside the flower was significantly higher before 11:00, significantly lower between 12:00 and 18:00, and did not differ significantly in blooming period (11:00) than temperature outside the flower (Figure 8a). The CV of temperature inside flower from 6:00 to 19:00 was 0.1088, which was significantly lower than which outside the flower (CV = 0.2884), indicating a better temperature stability inside the flowers.

During the whole monitoring period, the RH inside flower was higher than outside flower (Figure 8b). The CV of RH inside flower (CV = 0.1879) was smaller than outside flower (CV = 0.3594), indicating a more stable RH inside the flowers. These results indicated that floral closure could provide a more stable and higher RH inside the flowers (Figure 8b).

### 3.4 Effect of floral closure on seed production

**FIGURE 9:**
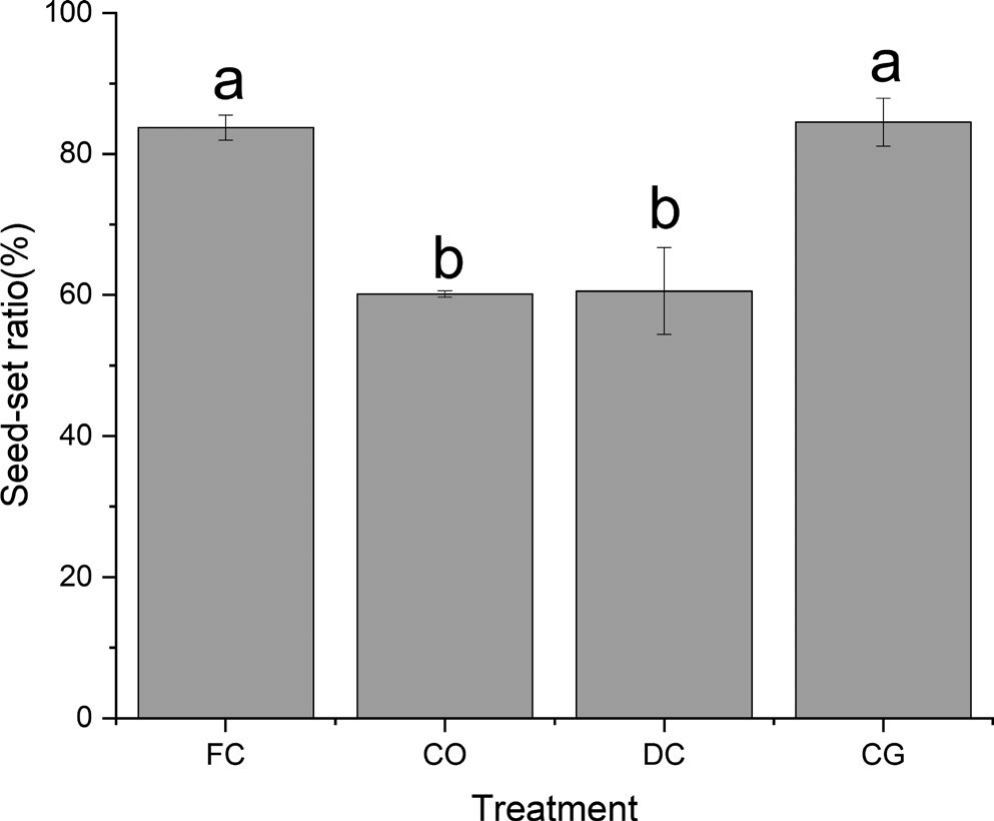
Seed‐set ratio (Mean ± SD) of *G*. *paludosa* under different treatments. Different letters within the figure indicate significant differences (*p* < .05). Bars indicate the standard deviations (*N* = 60). CG, control group; CO, compulsive openness; DC, delayed closure; FC, forced closure

The seed‐set ratio of forced closure flowers (83.72 ± 1.77%) has no difference with flowers under natural condition (84.51 ± 3.41%), indicating that forcing closure did not reduce the reproduction of *G*. *paludosa* (Figure 9). However, a sharp decline of seed‐set ratio occurred when flowers compulsive opened (60.13 ± 0.45%) and delay closed (60.57 ± 6.16%), indicating an over‐all decreased reproductive fitness when flowers of *G*. *paludosa* opened for a long time (*F* = 852.674, *p *< .001; Figure 9).

## 4 DISCUSSION

Repeatable floral closure, as commonly reported in several families, is considered as an important plasticity behavior in response to environmental conditions, especially in habitats with large diurnal temperature changes or high dew and rainfall (He et al., 2006; Von Hase et al., 2006), and/or flower early in spring (Abdusalam & Tan, 2014; Liu et al., 2017). Similarly, like the other species in Gentianaceae, *G*. *paludosa* exhibited an obvious repeatable floral closure. However, the rhythmic closure time was at noon, re‐opened in the next morning, representing a 7 h open and 17 h close rhythm (Figure 2), which was different with the other species of Gentianaceae (Bai & Kawabata, 2015; Bynum & Smith, 2001; He et al., 2006; Mu et al., 2010). As far as we know, this is the first report of the intermittent flower closure rhythm of Gentianaceae, which were mostly seen in Asteraceae and a few in Primulaceae (Brauner & Rau, 1966).

Temperature was apparently the most important exogenous factor which stimulated the floral closure of *G*. *paludosa*, including low and high temperature (Figures 5a and 6). Low temperature, as reported by many studies, was considered more related to the floral closure, especially in alpine area or early spring species (Abdusalam & Tan, 2014; Bynum & Smith, 2001; He et al., 2006; Liu et al., 2017; Mu et al., 2010). It is not difficult to understand because of at least two reasons. First, sudden temperature dropping caused by rain and thunderstorms in the QTP occurs frequently in July and August when *G*. *paludosa* is at its peak flowering period. Meanwhile, a low temperature‐induced floral closure can effectively protect the flower internal organs, such as anther and filament, style and stigma, from the damage of rain and thunderstorms, and also can prevent nectar diluting from rain (He et al., 2006; Huang et al., 2002). Second, low temperature may delay anther develop and slow pollen germination on stigmas while floral closure can provide a stable microenvironment that promoted pollen germination and avoided a decline in stigma receptivity (Abdusalam & Tan, 2014; Liu et al., 2017). However, high temperature‐induced temporal floral closure was seldom reported before, especially in alpine area, because conversed to high temperature, low temperature is considered as an important environmental limiting factor. Although numerous studies have proved that high temperature is harmful for the floral organ development, anther dehiscence, and pollen viability (Das et al., 2014; Wang et al., 2019; Yan et al., 2017), the effective adaptive strategies always focused on phytophysiological changing caused by genome modification (Ahanger et al., 2017; McClung & Davis, 2010; Wang et al., 2019), rather than morphological plasticity changes (except for Yan et al., 2017). We proved that a repeatable floral closure of *G*. *paludosa* could profit maintaining a suitable temperature range (Figure 8a), which, obviously, would protect the sex organs inside the flowers from damage of both low and high temperature, as well as provide a stable microenvironment. This is in line with previous findings showing *Anemone rivularis* could regulate the microenvironment inside the flowers to avoid damage by high temperature and radiation (Zhang et al., 2010).

Although numerous studies proved that floral closure was affected by light, including low and/or high light intensity (reviewed by Bai & Kawabata, 2015; van Doorn & Kamdee, 2014; van Doorn & van Meeteren, 2003), it seems not to be the case in alpine species belonging to Gentianaceae, such as *Gentiana straminea* (He et al., 2006), *Gentiana algida* (Bynum & Smith, 2001), and *Gentiana leucomelaena* (Mu et al., 2010). In this study, we did not detect a significant influence of illumination intensity on floral closure of *G*. *paludosa* (Figures 5b and 6). These observations are consistent with the findings of alpine species belonging to Gentianaceae. Besides much attention on temperature and light, the influence of relative humidity associated with flower movements have been explicitly investigated by only very few studies. It is probably because that most species tested did not react to changes in RH (except some nocturnal species), and changed RH was always mainly caused by the fluctuation of the temperature or light (van Doorn & van Meeteren, 2003; He et al., 2006). For *G*. *paludosa* in our study, continued high RH and medium RH could not delay or advance the time of floral closure, but low RH, both in one‐factor or two‐factor treatments, could apparently accelerate floral closure (Figures 5c and 6). This result suggested that low RH might be a selective pressure for floral closure of *G*. *paludosa*, which was confirmed by the more stable and higher RH inside the flowers compared with outside the flowers (Figure 8b). Closing advanced by low RH has not been reported before but, on the contrary, high RH or rain induced floral closure or floral traits changing were widely reported because of the benefit from protection of pollen grains in anthers and on stigmas and thus improving male and female fitness (Bynum & Smith, 2001; He et al., 2006; Huang et al., 2002; Kozuharova & Anchev, 2006; Mao & Huang, 2009; Sun et al., 2008; Von Hase et al., 2006; Wang et al., 2010). A reasonable explanation was high temperature, accompanied with a low RH, might induce temporal floral closure but it was seldom noticed in alpine species, and changed RH played a secondary role compared with temperature. This hypothesis was proved in various species of *Taraxacum* (Tanaka et al., 1987, 1988) and *Gentiana* (Bynum & Smith, 2001; He et al., 2006), and, as pointed by van Doorn and van Meeteren (2003), a rather complicated interaction exists between the influence of temperature, light, and relative humidity.

Both low/high temperature and low RH could stimulate floral closure of *G*. *paludosa* when blooming at 10:00, but all continued medium temperature could not influence the closure process when the flowers at a preclosing period (12:00). Additionally, re‐opening did not occur after all single‐factor and two‐factor treatments when flowers of *G*. *paludosa* full closed at 14:00. A strong endogenous rhythm of floral closure exists based on these results, that is, the floral closure of *G*. *paludosa* depended primarily on time. The reciprocal oscillations of opening and closing were quite different with other species in alpine area, especially Gentianaceae. For example, sensitive floral closure of some species belonging to *Gentiana* were dependent upon the magnitude of the temperature change regardless of the time of day (Bynum & Smith, 2001; Goldsmith & Hafenrichter, 1932; He et al., 2006), and flowers of *Eustoma grandiflorum* showed diurnal rhythms while opening in the morning and closing in the evening (Bai & Kawabata, 2015).

Although the physiological basis for this circadian rhythm remains unknown, the ecological function, based on our results, was manifested in a positive effect on reproduction, since floral closure of *G*. *paludosa* could significantly increase the seed‐set ratio (Figure 9). An interesting finding was that forcing closure did not reduce the reproduction of *G*. *paludosa* (Figure 9), suggesting an autonomous selfing. Accurately, delayed selfing, which is a flexible pollination mechanism assure seed production under the constraints of pollinator scarcity but ensure outcrossing when pollinators were available, occurs in *G*. *paludosa* (Duan et al., 2010). For *G*. *paludosa*, repeatable flower closure may ensure seed production by providing a relatively stable internal flower environment to protect the reproductive organs from rain (Bynum & Smith, 2001; van Doorn & van Meeteren, 2003; He et al., 2006), preventing pollination with pollen of low viability (Franchi et al., 2014; Zhang et al., 2014), promoting anther development and pollen function (Liu et al., 2017; Prokop et al., 2019; van Hase et al., 2006) and seed development (Clark & Husband, 2007; He et al., 2006). However, we failed to experimentally test the protective effect of this petal movement on the reproductive organs, and a further study is needed in the future. Furthermore, maintenance of flower opening and petal movement are resource cost, because petal movement always involves physiological changes, such as changes in epidermal cells (Wood, 1953), cell wall extensions (Brummel et al., 1999), carbohydrate metabolism (Bieleski et al., 2000), and hormone regulation (Reid et al., 1989). Thus, floral closure in the daytime might save resource that would allocate to maintenance of floral opening, which might enhance reproductive success of plant species capable of autonomous selfing.

Natural selection favors strategies that maximize reproductive fitness and, at the same time, reduce costs (Hirshfield & Tinkle, 1975; Williams, 1966). Physiological activities associated with flowering, such as nectar production, water balance, and respiration rate, are energy consuming (Ashman & Schoen, 1994, 1997; Castro et al., 2008; Nobel, 1977; Zhang et al., 2011). Repetitive closure is considered to reduce additional energy costs associated with flower maintenance to optimize reproductive success (Ashman & Schoen, 1997; van Doorn & van Meeteren, 2003). Considering that the cost of flower maintenance can be higher in high temperature environments (Caruso, 2006; Polowick & Sawhney, 1988; Prasad et al., 2008; Prokop et al., 2019, 2021; Ristic et al., 2007; Teixido & Valladares, 2013), flower closure in the early afternoon may be beneficial when the ambient temperatures are high. Furthermore, viability and germinability of pollen grains may also be compromised when the relative humidity is low, and closed petals could provide the optimum humidity to maintain pollen grains viability (Bahramabadi et al., 2018; van Doorn & van Meeteren, 2003; Fonseca & Westgate, 2005; Franchi et al., 2002, 2014; Nepi & Pacini, 1993). Therefore, flower closure appears to be beneficial for plants when daily temperature is high or relative humidity is low. In this case, high temperatures and low relative humidity after 14:00 in QTP may increase transpiration rates and decrease viability of reproductive organs, the endogenous rhythm of floral closure (closing begins at noon and a full‐closure occurs at early afternoon) is an adaptive strategy through shortening flower representation time to save the energy and protect reproductive organs against unfavorable environmental conditions. Furthermore, repeatable flower closure of *G*. *paludosa* may also ensure reproductive success by providing a more stable internal microenvironment.

## CONFLICT OF INTEREST

None declared.

## AUTHOR CONTRIBUTIONS

**Qinzheng Hou:** Conceptualization (equal); Data curation (equal); Formal analysis (equal); Funding acquisition (lead); Writing – original draft (lead); Writing – review & editing (equal). **Xiang Zhao:** Data curation (equal); Formal analysis (equal); Visualization (lead); Writing – original draft (supporting); Writing – review & editing (equal). **Xia Pang:** Data curation (supporting); Writing – original draft (supporting). **Meiling Duan:** Formal analysis (supporting); Visualization (supporting). **Nurbiye Ehmet:** Formal analysis (supporting). **Wenjuan Shao:** Visualization (supporting). **Kun Sun:** Conceptualization (equal); Data curation (equal); Formal analysis (equal); Writing – review & editing (equal).

## Data Availability

The raw data used in this study are available from the Dryad Digital. Repository: https://doi.org/10.5061/dryad.qv9s4mwg4.
